# Site Communication in Direct Formation of H_2_O_2_ over Single-Atom Pd@Au Nanoparticles

**DOI:** 10.1021/jacs.3c00656

**Published:** 2023-05-16

**Authors:** Rasmus Svensson, Henrik Grönbeck

**Affiliations:** Department of Physics and Competence Centre for Catalysis, Chalmers University of Technology, SE-412 96 Göteborg, Sweden

## Abstract

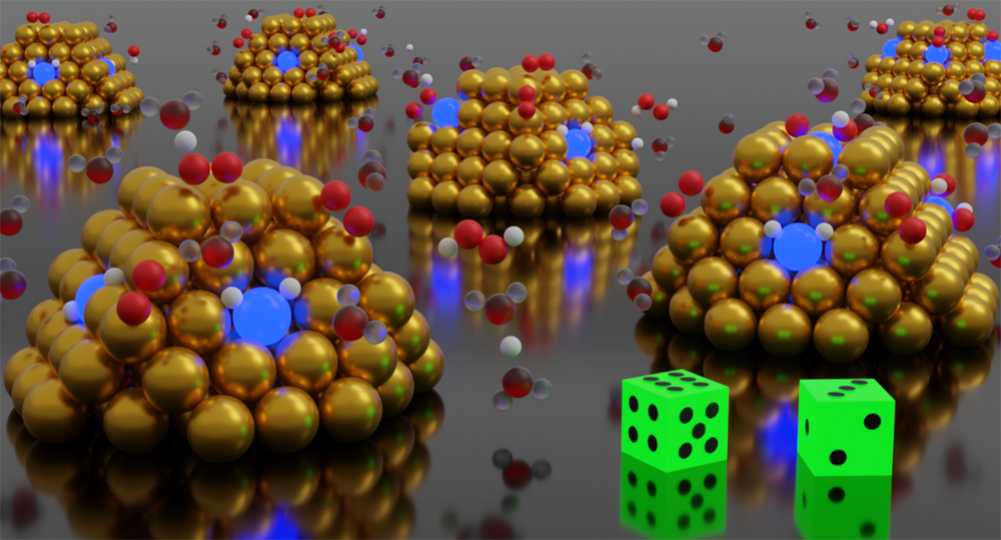

Single atom alloy catalysts offer possibilities to obtain turnover frequencies and selectivities unattainable by their monometallic counterparts. One example is direct formation of H_2_O_2_ from O_2_ and H_2_ over Pd embedded in Au hosts. Here, a first-principles-based kinetic Monte Carlo approach is developed to investigate the catalytic performance of Pd embedded in Au nanoparticles in an aqueous solution. The simulations reveal an efficient site separation where Pd monomers act as active centers for H_2_ dissociation, whereas H_2_O_2_ is formed over undercoordinated Au sites. After dissociation, atomic H may undergo an exothermic redox reaction, forming a hydronium ion in the solution and a negative charge on the surface. H_2_O_2_ is preferably formed from reactions between dissolved H^+^ and oxygen species on the Au surface. The simulations show that tuning the nanoparticle composition and reaction conditions can enhance the selectivity toward H_2_O_2_. The outlined approach is general and applicable for a range of different hydrogenation reactions over single atom alloy nanoparticles.

## Introduction

Hydrogen peroxide (H_2_O_2_) is an important industrial chemical with mild oxidizing properties and low environmental impact with only H_2_O and O_2_ as side products during oxidation.^1^ The area of usage ranges from bleaching and wastewater treatment to organic synthesis.^2−4^ H_2_O_2_ is currently produced by sequential reduction and oxidation of anthraquinones.^1^ The anthraquinone process has a high selectivity toward H_2_O_2_; however, it requires large-scale facilities, is energy demanding, involves complicated liquid–liquid extractions, and produces toxic wastes. It is therefore desirable to replace the anthraquinone process with a process where H_2_ and O_2_ react directly to form H_2_O_2_. A direct process has the potential to be operated at small scale and to be cost and energy efficient.^5,6^

**Figure 1:**
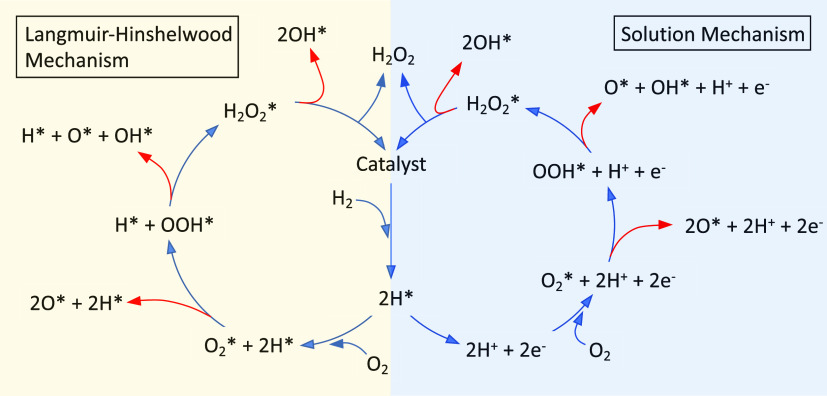
Proposed mechanisms for direct formation of H_2_O_2_ from O_2_ and H_2_ over metal nanoparticles. The left cycle shows H_2_O_2_ formation via a Langmuir–Hinshelwood mechanism, whereas the right cycle shows H_2_O_2_ formation via the solution or ligands.

Direct synthesis of H_2_O_2_ occurs via sequential addition of hydrogen to O_2_. Scission of the O–O bonds leads to irreversible formation of water and should be avoided.^7,8^ Different mechanisms for direct H_2_O_2_ formation over metal nanoparticles (NPs) have been discussed in the literature.^9,10^ The mechanisms are sketched in Figure 1. The left reaction cycle shows H_2_O_2_ formation via a Langmuir–Hinshelwood mechanism. H_2_ in this mechanism is adsorbed dissociatively, forming atomic H* on the surface. H* is added sequentially to molecularly adsorbed O_2_ forming H_2_O_2_. The right reaction cycle shows H_2_O_2_ formation where protons are transferred to the adsorbed O_2_ via the solution^9^ or alternatively ligands coating the metal NP.^11^ It is clear that a combination of the two mechanisms could apply. The side reactions (red arrows) lead to the unwanted formation of water, as O–O scission is virtually irreversible. Given the reaction cycles, a desirable catalyst for direct H_2_O_2_ formation should dissociate H_2_ and interact strongly enough with O_2_ to promote adsorption without O–O bond rupture. Different transition metal catalysts have been investigated for direct H_2_O_2_ formation, including Pd^7,12,13^ and Au^5,14,15^ NPs. High rates for H_2_O_2_ formation have been reported over Pd catalysts, however, with low selectivity due to the irreversible O–O bond scission and the subsequent water formation.^5,15,16^ Thus, the interaction between Pd and oxygen is too strong. One possibility to reduce the interaction between Pd and oxygen is to coat the metal with ligands.^11^ Another option to enhance the selectivity is to alloy Pd with a less reactive metal such as Au. Experiments have shown that it is possible to obtain high yields and high selectivity for H_2_O_2_ by alloying Pd with Au.^15,17^ The reason for the improved selectivity compared to Pd has been assigned to the suppression of active sites^5^ and geometric effects.^18^ It has recently been shown that selectivities approaching 100% can be obtained in the dilute limit of single Pd monomers embedded in a Au host.^18^ The Pd–Au alloy composition generally depends on the manufacturing method.^15,19,20^ In the case of dilute alloys, the distribution of Pd monomers has been measured to be close to random with a large fraction residing close to Au edges.^20^

The mechanistic understanding of hydrogen transfer reactions over dilute alloys of Pd monomers in Au (Pd@Au) is based on density functional theory calculations performed on extended surfaces.^18,21−23^ These studies have explored reaction paths over the Pd monomers and the possibility to enhance the selectivity by embedding one active site in an inert surface. However, technical catalysts are based on supported NPs, which differ with respect to extended surfaces, as they include a large number of different sites. (Roughly 15% of the surface atoms on a typical 7 nm particle are edge and corner sites.) Understanding the reaction mechanisms and the kinetic behavior over nanoparticles would potentially enable design of catalysts with improved performance. Here, we investigate the direct formation of H_2_O_2_ from O_2_ and H_2_ over Pd@Au nanoparticles, which are compared with extended surfaces. The potential energy landscapes for the reaction over a range of different Au and Pd@Au systems are mapped by density functional theory calculations, taking the effect of an aqueous solution into account. The reaction kinetics over NPs are modeled using scaling relation kinetic Monte Carlo simulations including both the Langmuir–Hinshelwood mechanism and solution mechanism (Figure 1). The kinetic Monte Carlo simulations are used to identify the dominant reaction mechanism as a function of NP composition and reaction conditions. We find that Pd monomers embedded in Au(111) have a close to 100% selectivity toward H_2_O_2_. Dissociated H on the metal surface undergoes an exothermic redox reaction with water to form water-solvated hydronium ions over a negatively charged metal surface. The presence of undercoordinated Au sites on NPs has a positive effect on the selectivity thanks to an efficient separation of sites that dissociate H_2_ (Pd) and adsorb O_2_ (Au). The facile intersite communication makes the selectivity over NPs sensitive to the number and distribution of Pd monomers. The simulated kinetic behavior is in good agreement with available experimental data and uncovers mechanisms that could be used to further improve the catalytic performance of dilute alloys. The simulations stress the need to account for the complete potential energy landscapes of solvated NPs to properly describe the kinetic behavior. The outlined approach is general and applicable for a range of different hydrogenation reactions.

## Computational Approaches

### Electronic Structure Calculations

Electronic structure calculations are performed using the Vienna Ab initio Simulation Package (VASP).^24−28^ The interaction between the core and valence electrons is described using the projector augmented-wave method.^28,29^ The considered valence electrons are 1s^1^ for H, 2s^2^2p^4^ for O, 5s^0^4d^10^ for Pd, and 6s^1^5d^10^ for Au. The exchange–correlation functional proposed by Perdew, Burke, and Ernzerhof^30^ is used together with the Grimme-D3 correction,^31,32^ to capture the van der Waals interactions. The plane waves are truncated at an energy of 450 eV in the expansion of the Kohn–Sham orbitals. Energies are considered to be converged when the change in electronic energy and Kohn–Sham eigenvalues between two succeeding electronic iterations is smaller than 1.0 × 10^–5^ eV. Structures are regarded to be converged when the forces on all cores are smaller than 0.03 eV/Å. The lattice constant and cohesive energy of Au are calculated to be 4.10 Å and −3.69 eV, respectively, which is in good agreement with the experimental results of 4.08 Å and −3.79 eV, respectively.^33^

To model the surfaces and NPs, adsorption energies, transition state energies, and vibrational modes are calculated using (3 × 3) surface cells for Pd@Au(111), Pd@Au(100), Pd@Au(211), and Au(211). The lattice constant of Au is used for the highly diluted Pd@Au systems.^34^ The surfaces are described by four atomic layers where positions of atoms in the two bottom layers are kept fixed to emulate a bulk system. The surface slabs are separated by 14 Å of vacuum. The Brillouin zone for the surface calculations is sampled with a (7 × 7 × 1) *k*-point mesh using the Monkhorst–Pack scheme.^35^ The reference energies and vibrational modes of gaseous O_2_ and H_2_ are calculated using a (14, 15, 16) Å box. The solvation energies of H_2_O and H_2_O_2_ are calculated using a water-filled cubic box with length 8.43 Å and a H_2_O density of 1 g/cm^3^. The vibrational modes are approximated as harmonic oscillators and calculated using finite differences. Transition states for the considered elementary reactions are obtained using the climbing image nudged elastic band (CI-NEB) method.^36,37^

To capture the influence of the aqueous solution, a water layer is added to all surface–adsorbate structures. Structures are relaxed from the lowest energy configurations originating from a 5 ps molecular dynamics simulation performed at 300 K. Adsorption energies are in this case calculated with respect to the surfaces with a water layer. Constrained molecular dynamics is performed to investigate the transfer of protons from the surface to the solution (Figure 1).

### Kinetic Monte Carlo Simulations

The kinetic behavior of direct H_2_O_2_ formation over NPs and extended surfaces is explored using kinetic Monte Carlo (kMC) simulations. The kMC method is a stochastic approach, which allows for explicit consideration of different types of catalytic sites.^38−40^ The possibility to treat different sites is crucial to describe reactions over alloy particles, with different structural and chemical sites.

The considered reaction events (Figure 1) for H_2_O_2_ and H_2_O formation are
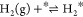
R1
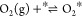
R2
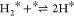
R3
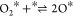
R4
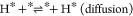
R5
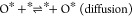
R6
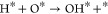
R7
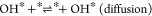
R8
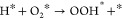
R9
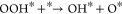
R10
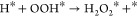
R11
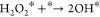
R12
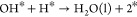
R13
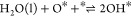
R14
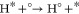
R15
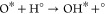
R16
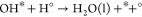
R17
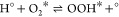
R18
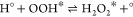
R19
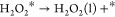
R20In the reaction network, * and ° denote a surface and solution site, respectively. The kMC simulations are performed using coarse-grained sites; that is, one combined geometric site is used to describe top, bridge, and hollow sites. For the extended surfaces, the turnover frequency and selectivity are simulated using a 20 × 20 lattice with periodic boundary conditions. The reactions over nanoparticles are simulated using truncated octahedra, with diameters of about 2.7 nm.

The kMC simulations are performed using the first reaction method.^41,42^ In the first reaction method, a list of time of occurrences, *t*_β,α_, is calculated for all possible events that transfer the system from a current state α to a future state β. The time of occurrence is calculated according to
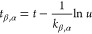
1where *k*_β,α_ is the rate constant of the considered event and *u* is a random number from a uniform distribution in the interval (0, 1). The time and system are evolved by performing the event with the shortest time of occurrence. After the execution of an event, time of occurrences are calculated for events that are enabled, whereas events that are disabled are removed from the list of events.

Adsorption steps are considered to be barrierless with rate constants described by collision theory:
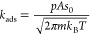
2where *p* is the partial pressure of the gas, *A* is the area of the adsorption site, and *s*_0_ is the sticking coefficient at zero coverage. Experimental results for Pd-only systems are used to approximate the sticking coefficients of H_2_ and O_2_ on the Pd monomers. The sticking coefficients have been measured to be similar for the two gases, being 0.6 on Pd(111)^43−45^ and 0.8 on Pd(100).^45−47^ The sticking coefficient for Pd(100) is used also for Pd@Au(211).

Thermodynamic consistency for reversible reactions is ensured by calculating the desorption rate constant from the equilibrium constant.
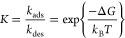
3where Δ*G* is the Gibbs free energy difference between initial and final states. The reaction rate constants are calculated from transition state theory:
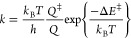
4where Δ*E*^⧧^ is the zero-point-corrected energy difference between the transition state and the initial state. *Q*^⧧^ and *Q* are the partition functions for the transition state (excluding the reaction coordinate) and the initial state, respectively. For the gas phase molecules, the partition function is expressed as the product between the translational, rotational, and vibrational partition functions. The partition functions for adsorbed species are treated as frustrated vibrations, i.e.,
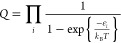
5where ε_*i*_ is the energy of vibrational mode *i*. Modes with energies lower than 12.4 meV (100 cm^–1^) are set to 12.4 meV due to the anharmonic behavior and computational uncertainties of such modes.

The considered reaction network for direct formation of H_2_O_2_ involves events and sites that require special attention, namely, the influence of the aqueous solution, the description of reactions over undercoordinated sites, and events that are very fast. The elementary reactions involved in the solution mechanism (Figure 1)
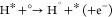
6are studied using constrained molecular dynamics (* and ° denote a surface and a solution site, respectively). The mechanism involves proton desorption from the surface with the subsequent formation of a hydronium ion in the water layer and an excess electron in the surface. The molecular dynamics simulations reveal that the barrier for the proton desorption is low (see Supporting Information (SI)), which is in agreement with previous calculations for proton transfer to small H_2_O clusters on Au(111).^48^ The rate for proton desorption is therefore dominated by the probability of the water layer to be oriented in a way that can accommodate the desorbed proton. The structural relaxation time of water close to a metal surface is about 2 ns,^49^ which is considerably slower than the structural relaxation time in bulk water (∼5 ps). The prefactor of the proton–electron transfer is, thus, set to 0.5 × 10^9^ s^–1^. The reaction network contains several steps that involve water restructuring (R13–R20). The prefactors for all proton transfer reactions are set to 0.5 × 10^9^ s^–1^, whereas the prefactor for H_2_O_2_ desorption is set to 1 × 10^9^ s^–1^. Equation 3 is used to maintain thermodynamic consistency also in these reactions. The existence of protons in the water solution is accounted for using an explicit site (° in the reaction network). As the diffusion of protons in water is significantly faster than all considered events,^50^ the explicit solution site is connected to all surface sites. The solution is set to have pH = 7, which limits the number of excess protons in the solution. Over the periodic (3 × 3) surface, desorption of one proton to the solution is exothermic, whereas the desorption of a second proton is endothermic when the water is described by one layer. We limit the number of excess protons in the solution to four in the kMC simulations over the (20 × 20) surface lattice and the NPs. The results are not sensitive to the allowed number of excess protons, as demonstrated by explicit simulations for Pd embedded in a (111) facet of an NP allowing for two and eight excess protons.

Scaling relations based on generalized coordination numbers are used to describe the energetic landscape for O_2_, O, OH, and OOH species on Au NPs.^51,52^ The generalized coordination number^51^ of atom *i* is defined as^52^
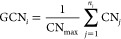
7where *n*_*i*_ is the number of nearest neighbors, CN_*j*_ is the coordination number of neighbor *j*, and CN_max_ is the maximum coordination number for the specific atom or group of atoms. The scaling relations for the adsorption energies are obtained by considering a series of model structures. A complete list of model structures, generalized coordination numbers, and adsorption energies is given in the SI.

A general complication in kMC simulations is the large separation in rates for different reactions. For direct H_2_O_2_ formation over Pd@Au nanoparticles, the O_2_ adsorption and desorption events are considerably more frequent than other events. The adsorption/desorption of O_2_ is therefore treated in a probabilistic mean-field approach. The O_2_ coverage is described implicitly, where empty Au sites are assigned an occupancy based on the equilibrium constant for the considered site. The probability (coverage) of having O_2_ adsorbed on a specific site is in this approach given by
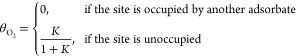
8

## Results and Discussion

The considered Pd@Au NPs are systems with a range of different sites and consequently a complex potential energy landscape. Different strategies are therefore used to describe the potential energy landscape. The adsorption and transition state energies are calculated explicitly over Pd monomers embedded in the Au host, whereas the complete energy landscape over Au NPs is described using scaling relations based on generalized coordination numbers.^51^ The use of scaling relations is an efficient approach to obtain the potential energy landscape for the multitude of different sites on an NP.^53^

### Potential Energy Landscapes for H_2_O_2_ Formation

**Figure 2:**
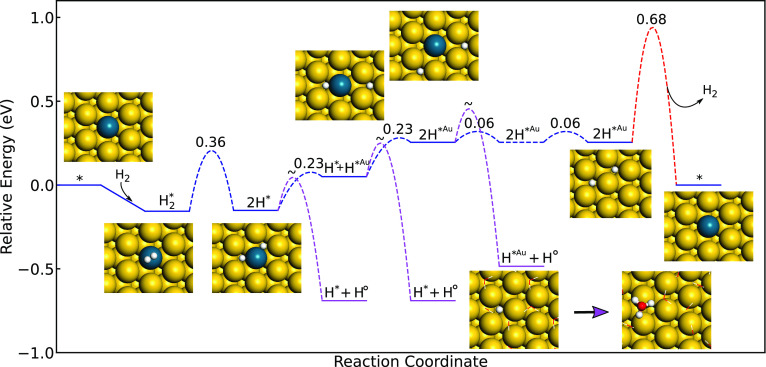
Reaction mechanism for hydrogen dissociation and diffusion over Pd@Au(111). The corresponding adsorption and dissociation energies over Pd@Au(100) and Pd@Au(211) are shown in the SI. * denotes a surface site, and H° denotes a solvated proton.

The first crucial step in the reaction is H_2_ dissociation and H diffusion. The corresponding potential energy landscape over Pd@Au(111) is shown in Figure 2. H_2_ adsorbs with an adsorption energy of 0.16 eV. Dissociation into atomic H residing in fcc-hollow sites is associated with a barrier of 0.36 eV. The dissociated H may thereafter diffuse away from the Pd monomer, with a barrier of 0.23 eV, or undergo a strongly exothermic redox reaction, where a proton desorbs into the water solution while leaving a delocalized excess electron on the surface. The energetics of the redox reaction depends weakly on whether the process occurs close to or far from a Pd monomer [see SI for desorption from Au(111), Pd@Au(111), and Pd(111)]. The diffusion barrier for H over the Au(111) surface is 0.06 eV. Despite the fact that adsorbed H is endothermic with respect to gas phase H_2_, H–H association is associated with a high barrier (0.68 eV). H will therefore remain on the Au(111) surface at relevant temperatures or desorb as a proton to the water solution. The only viable path for H_2_ desorption is recombination over the Pd monomers. The state with a proton in the solution (in the form of H_3_O^+^) and an excess electron on the surface is exothermic and accessible because of the facile H_2_ dissociation over Pd monomers, the low adsorption energy of H, and the high electronegativity of Au(111). As the process is exothermic, the metal particles are slightly negatively charged during the reaction. This is in agreement with experimental findings where the reaction is reported to produce a reducing potential for metal particle catalysts.^54^

**Figure 3:**
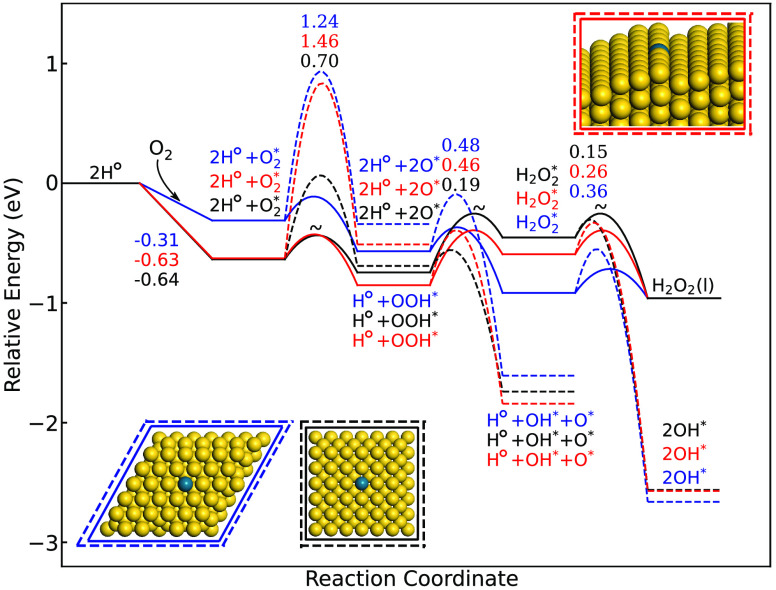
Potential energy landscape for the formation of H_2_O_2_ and side reactions forming H_2_O over Pd@Au(111) (blue), Pd@Au(100) (black), and Pd@Au(211) (red). * denotes a surface site and H° denotes a solvated proton.

The potential energy landscapes for H_2_O_2_ formation over Pd embedded in Au(111), Au(100), and Au(211) via the solution route are shown in Figure 3. The corresponding energies for H_2_O_2_ formation via the Langmuir–Hinshelwood mechanism over Pd@Au(111) and Pd@Au(211) are reported in the SI.

The reference state in Figure 3 is a bare surface with two protons in the solution (2H°). The adsorption energy of O_2_ depends on whether H* is adsorbed at the Pd site, and we consider in Figure 3 the case with zero H* coverage. The adsorption of O_2_ is influenced by the presence of water, which facilitates a metal to O_2_ charge transfer (see the SI). The dissociation of  is prevented by high barriers. The formation of OOH* is exothermic over all three surfaces. The subsequent formation of  is, however, only exothermic over Pd@Au(111). Dissociation of OOH* is competitive with the endothermic formation of  over Pd@Au(100) and Pd@Au(211). Note that the formation of OOH* and  are reversible, owing to the high stability of the proton in the solution. This is in variance with the Langmuir–Hinshelwood path (SI), where both OOH* formation and  formation are exothermic and the barriers for the backward reactions are substantial. The potential energy landscapes for Pd@Au(100) and Pd@Au(211) have local minima at OOH* and a proton in the solution, which is preferred with respect to . Thus, the potential energy landscapes indicate that the selectivity is lower over Pd@Au(100) and Pd@Au(211) compared to Pd@Au(111).  desorption competes with irreversible  dissociation forming 2OH*. The dissociation barrier is highest on Pd@Au(111) and lowest on Pd@Au(100). We find that the potential energy landscapes over Pd@Au(100) and Pd@Au(211) are similar; both systems bind , H*, , and OOH* stronger than does Pd@Au(111) and have lower barriers for OOH* and  dissociation.

**Figure 4:**
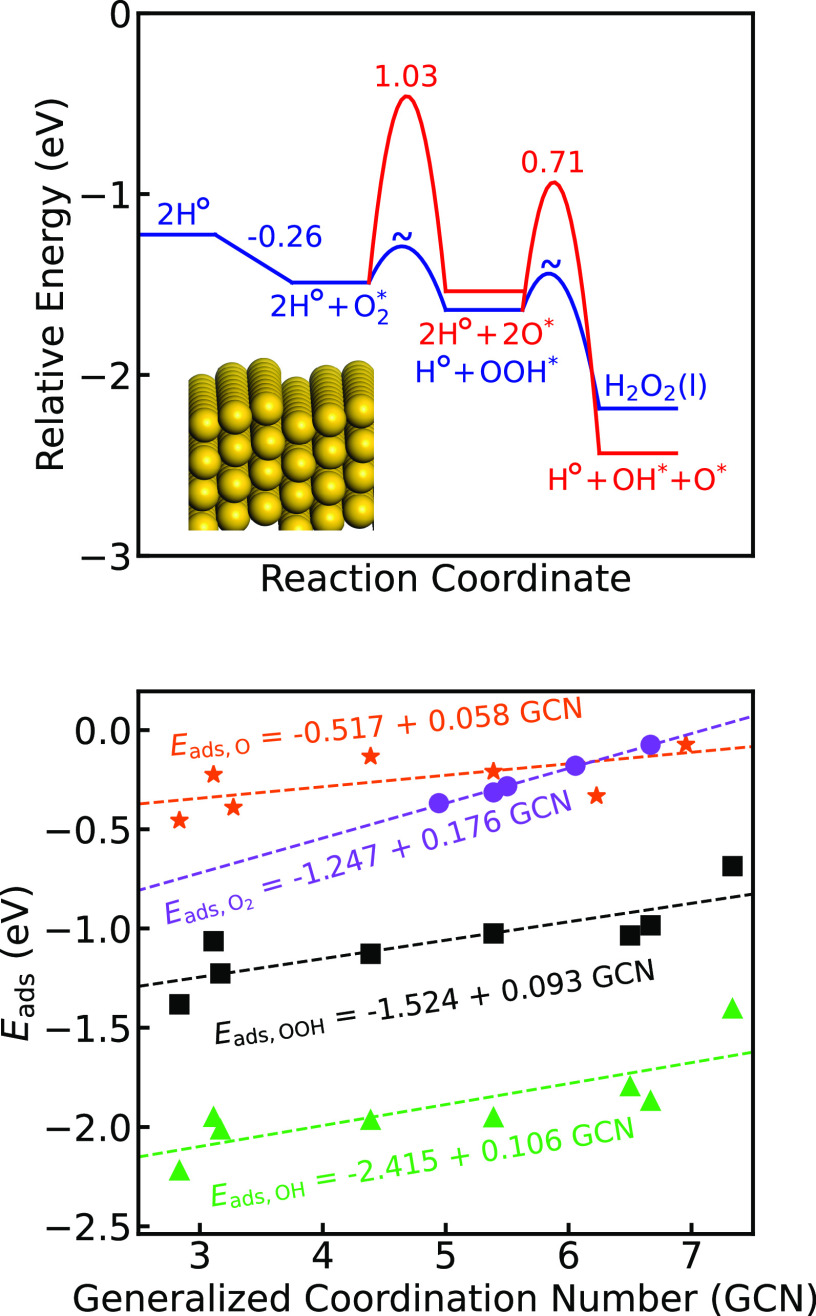
Top: The potential energy landscape for the formation of H_2_O_2_ and competing side reactions over Au(211). * denotes a surface site and H° denotes a solvated proton. Bottom: Non-zero-point-corrected scaling relations to describe adsorption energies on undercoordinated Au NP sites. The scaling relations have mean absolute errors of 0.086 eV for O*, 0.0062 eV for , 0.11 eV for OH*, and 0.082 eV for OOH*.

Owing to the facile H diffusion over gold surfaces (H*) and proton diffusion via the water solution (H°), it is possible to form OOH* and H_2_O_2_ from adsorbed O_2_ on undercoordinated Au sites. Scaling relations are used to describe the energy landscape over Au sites. The potential energy landscape for H_2_O_2_ formation over Au(211) together with scaling relations are shown in Figure 4. The dissociation barriers of  and OOH* are high, and  desorbs once formed. The formations of OOH* and H_2_O_2_ are both exothermic over Au(211), which indicates that the reaction could occur on undercoordinated sites. The exothermicity in the formation of the intermediates changes with generalized coordination number, as the scaling is different for  and OOH*.

### Kinetic Monte Carlo Simulations

Kinetic Monte Carlo simulations are used to investigate turnover frequencies (TOF) and selectivities (*S*) for direct H_2_O_2_ formation over different Pd@Au structures. Simulations are performed to explore the effects of reaction conditions (temperature and pressure) as well as the effect of the number and placement of Pd monomers. The TOF is defined as the number of H_2_O_2_ (or H_2_O) formed per Pd monomer and second. The reaction takes place in a water solution, which implies that adsorption of H_2_O on the Pd monomer may block the site for H_2_ and O_2_ adsorption. The adsorption and desorption of H_2_O on the Pd site are fast processes, which we include by scaling the TOFs with a Boltzmann distribution determined by the adsorption energy of H_2_O on the Pd monomer from the water solution. The selectivity is given by
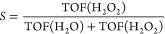
9

#### Influence of Temperature

**Figure 5:**
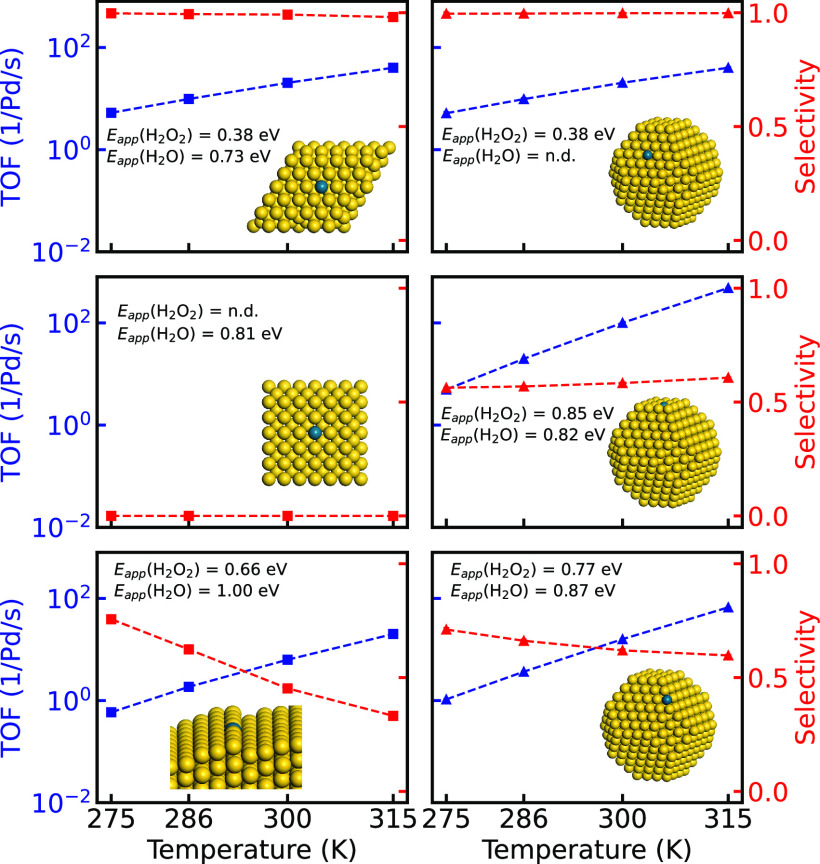
Turnover frequency of H_2_O_2_ (blue) and selectivity (red) as a function of temperature, in the interval 275–315 K over Pd@(111), Pd@100), Pd@(step), Pd@NP(111), Pd@NP(100), and Pd@NP(edge). The partial pressures of H_2_ and O_2_ are set to 100 kPa. The standard deviations are taken over 30 independent simulations.

The temperature dependence of TOFs and selectivities is shown in Figure 5 for Pd embedded in Au(111), Au(100), and Au(211), as well as the corresponding positions in a 2.7 nm truncated octahedron. We denote the systems with extended surfaces Pd@(111), Pd@(100), and Pd@(211), whereas the NP systems are denoted Pd@NP(111), Pd@NP(100), and Pd@NP(edge). The partial pressures of H_2_ and O_2_ are set to 100 kPa for all simulations in Figure 5.

The selectivity over Pd@(111) is close to 100% in the entire temperature interval, which is also the case for a Pd monomer placed in the Au(111) facet of the truncated octahedron [Pd@NP(111)]. Importantly, however, the mechanism for H_2_O_2_ formation over Pd@(111) is significantly different compared to Pd@NP(111). H_2_O_2_ is on Pd@(111) formed exclusively over the Pd monomer, whereas more than 95% of H_2_O_2_ is formed over undercoordinated Au edge and corner sites in the case of Pd@NP(111). The reason for similar TOFs is that H_2_ dissociation occurs only on the Pd monomer, which has the same properties on Pd@(111) and Pd@NP(111). The high selectivity for the two systems is in both cases thanks to a high OOH* dissociation barrier.

Placing Pd in the extended (100)-surface has dramatic effects on the H_2_O_2_ formation. The TOF and selectivity toward H_2_O_2_ is zero for Pd@(100), which is a consequence of facile OOH* and  dissociation. A reasonable selectivity (around 60%) is instead predicted for Pd@NP(100), despite the fact that the selectivity is zero for Pd@(100). The difference between the extended surface and the NP is again traced to different mechanisms. H_2_O_2_ is for the NP formed via the solution mechanism over undercoordinated Au sites, which have considerable barriers for OOH* dissociation. H_2_O is on Pd@NP(100) instead formed over the Pd site; thus the two products are formed over different sites. The selectivity is not as high as on Pd@NP(111) because of the reversibility of the OOH* formation on the undercoordinated Au sites; the barrier for OOH* decomposition to  and H° is low.

To investigate the effect of placing the Pd monomer in a step, we consider Au(111) with a Au(211) step [Pd@(step)]. Despite similar potential energy landscapes over the Pd monomer embedded in Au(100) and Au(211) (see Figure 3), the kinetic behavior is different. The selectivity is about 50% over Pd@(step), which is a consequence of undercoordinated Au sites where O_2_ can adsorb and form H_2_O_2_. The reason for the limited selectivity is the low coverage and concentration of H; thus OOH* may decompose to OH* and O* before  is formed. The performance of Pd@NP(edge) is similar to Pd@(step), and the solution mechanism is the dominant reaction path. Despite that the number of undercoordinated Au sites is much higher on the NP compared to Pd@(step), the selectivity on the Pd@NP(edge) is not markedly higher than on the Pd@(step). A similar selectivity arises as the OOH* decomposition to  and H° is more facile on the NP edges and corners than on the Pd@(step).

#### Influence of Pressure

**Figure 6:**
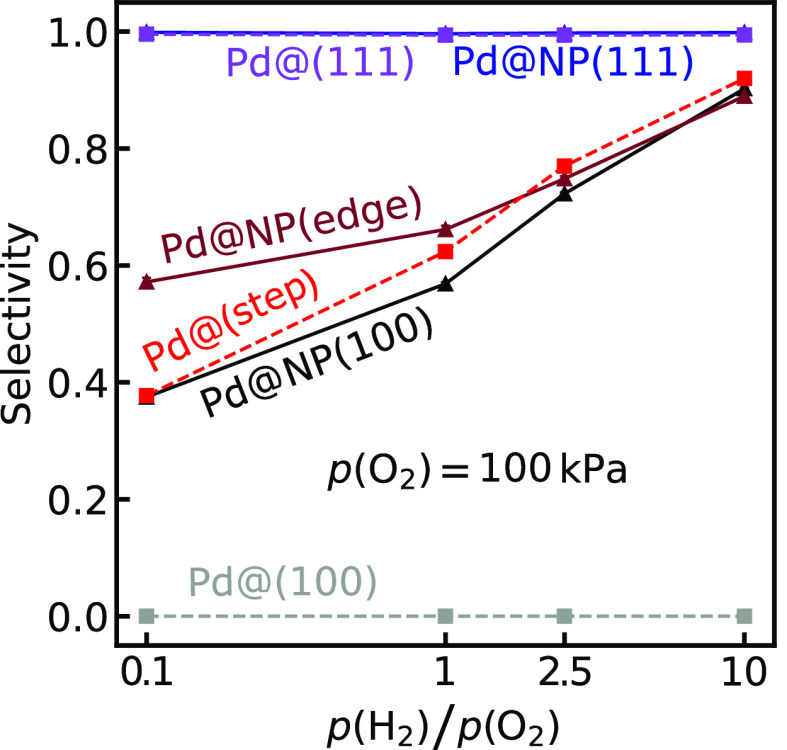
Selectivity toward H_2_O_2_ as a function of *p*(H_2_)/*p*(O_2_) ratio, over Pd@(111), Pd@(100), Pd@(step), Pd@NP(111), Pd@NP(100), and Pd@NP(edge). All simulations are performed at 286 K. The standard deviations are taken over 30 independent simulations.

Besides temperature, the kinetic behavior could be modified with the reactant pressures. The selectivities over the different structures as a function of *p*(H_2_)/*p*(O_2_) ratio are shown in Figure 6.

For Pd@(111) and Pd@NP(111), the selectivity is close to 100%, with a weak dependence on *p*(H_2_)/*p*(O_2_) ratio. The high selectivity is connected to the large barriers for O–O bond rupture. For Pd@(111), H_2_O_2_ is formed exclusively over the Pd monomer, whereas H_2_O_2_ is formed both over Pd@Au(111) and undercoordinated Au sites over Pd@NP(111).

Similar to the temperature dependence, the selectivity is close to zero over a Pd monomer located in the extended Au(100) surface regardless of the partial pressure ratio. The reason for the low selectivity is the high rate for OOH* dissociation compared to the formation rate of . The ratio of the partial pressures has, instead, an effect when the Pd monomer is placed in a (100) facet of an NP. An alternative reaction route is enabled on the NP, as H_2_O_2_ can form over undercoordinated Au edge and corner sites, where the barriers for O–O scission are higher than over the Pd@Au(100). The selectivity approaches 100% at high *p*(H_2_)/*p*(O_2_) ratio, as H_2_ excess effectively blocks the Pd monomer and prevents O_2_ adsorption, hence hindering OOH* formation and dissociation. Moreover, a high H_2_ pressure increases the amount of water-solvated protons in the system (the solution site has a coverage of about 10^–3^ per NP for NPs where the Pd monomers are located in the (111) facets), which results in shorter lifetimes of OOH* and higher H_2_O_2_ formation rates. The increased concentration of protons in the solution at higher H_2_ pressures agrees with experimental findings for the reaction over PtAu_60_ NPs.^54^

The dependence on the pressure ratio for Pd@(step) and Pd@NP(edge) is similar to Pd@NP(100); an increased *p*(H_2_)/*p*(O_2_) ratio yields higher selectivity. The improved selectivity with H_2_ excess is again thanks to hydrogen blocking of the Pd monomer, which suppresses O_2_ adsorption on the Pd site. The difference in selectivity for Pd@(step) and Pd@(100) is not directly evident from the potential energy landscapes for the two Pd monomers in Figure 3. The underlying reason is that Pd@(step) contains undercoordinated Au sites where H_2_O_2_ can be formed; hence the formation of H_2_O_2_ on Pd@(step) is not limited to the Pd monomer as is the case for Pd@(100). Generally, the selectivity toward H_2_O_2_ is favored by high H_2_ and low O_2_ pressures.

#### Effect of Multiple Pd Monomers

**Figure 7:**
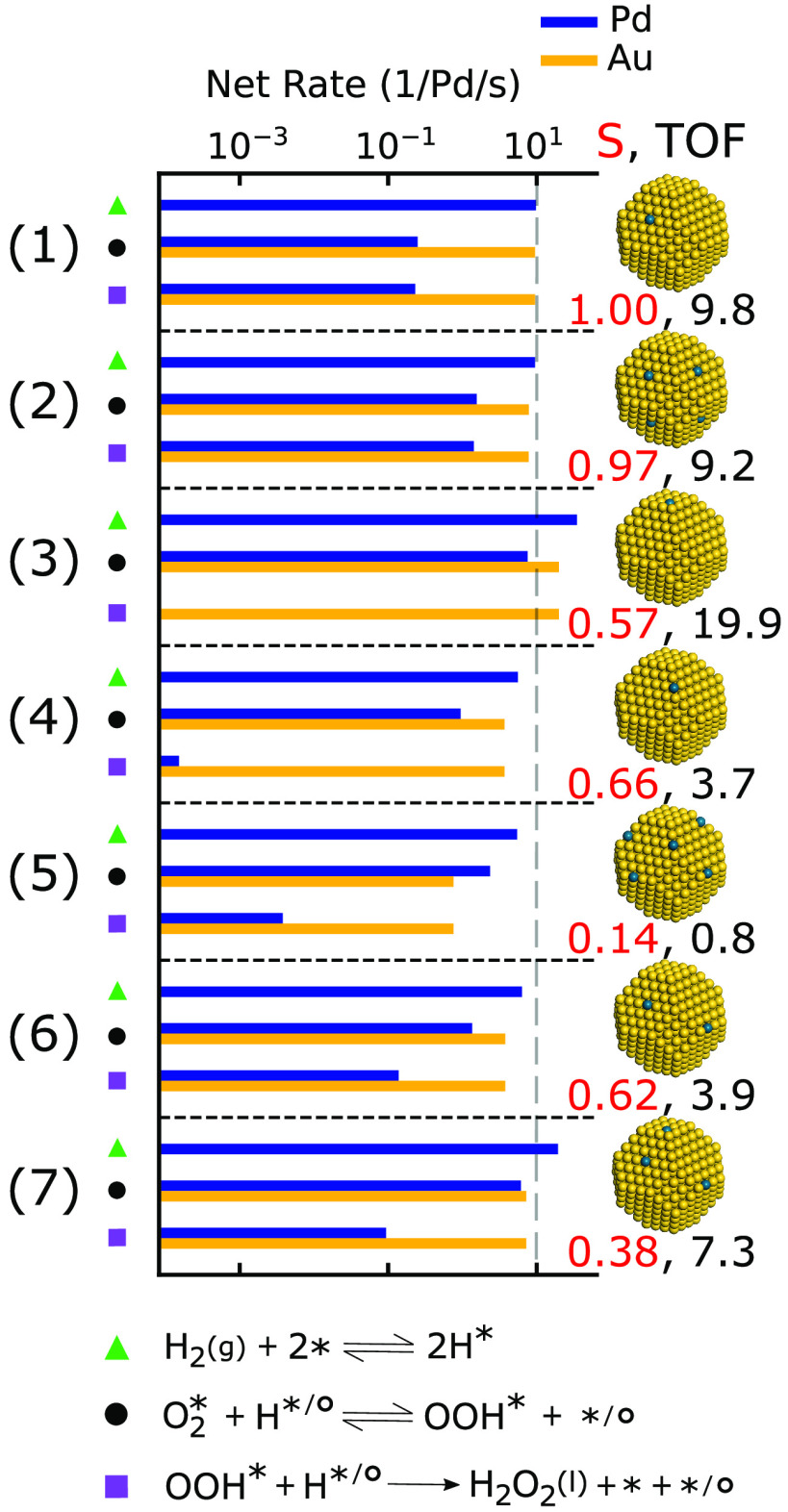
Steady state net rates of H_2_ dissociation, OOH* formation, and H_2_O_2_ desorption, as well as the selectivity over truncated octahedra with different Pd distributions. All simulations are performed at 286 K and O_2_ and H_2_ pressures of 100 kPa.

To elucidate the governing mechanisms for H_2_O_2_ selectivity and the effect of the number and location of Pd monomers, the steady state net rates over Au and Pd sites for selected reaction steps are presented in Figure 7. The considered NP is a 2.7 nm truncated Au octahedron with Pd monomers placed in (111) and (100) facets as well as in edges. H_2_ dissociation determines the TOF over the NP, OOH* formation shows where the reaction occurs, and H_2_O_2_ formation determines the selectivity and TOF toward H_2_O_2_.

The selectivity over a truncated octahedron with a single Pd monomer is clearly affected by the position of the monomer (compare with Figure 5 and Figure 6), where the selectivity toward H_2_O_2_ is close to 100% over Pd@NP(111) and considerably lower when Pd is located at either Au(100) or on an NP edge. The locations of the Pd monomers become even more important when the number of Pd monomers is increased. When one Pd monomer is placed in each (111) facet, the selectivity is still close to 100%. The situation is different with many Pd monomers located in edge sites, where the selectivity toward H_2_O_2_ is severely decreased with respect to the case with only one Pd monomer. For NPs with Pd monomers located at (111), (100), and edge sites, the selectivity toward H_2_O_2_ is determined by the Pd monomers in (100) and edge sites.

For an NP with a single Pd monomer in a (111) facet, H_2_ is dissociated over the Pd monomer. Owing to the facile H diffusion, the net formation rates of OOH* and H_2_O_2_ are dominated by reactions over the undercoordinated Au sites. As the selectivity is almost 100%, the steady state net formation rates of OOH* and H_2_O_2_ are equal to the net rate of H_2_ dissociation. For an NP where one Pd monomer is placed in each (111) facet, the Au-edge/Pd ratio is decreased, which leads to a higher OOH* net formation rate over the Pd monomers. However, the net formation rate of H_2_O_2_ desorption is only slightly reduced compared to the net rate of OOH* formation over both Au and Pd, thanks to the high selectivity over Pd@Au(111).

When the Pd monomer is placed in either the (100) facet or in an edge, the net formation rate of H_2_O_2_ is lower than the net rate of H_2_ dissociation; thus the selectivity is low. In these cases, the net rates of OOH* formation over Pd are similar to the net formation rates over Au. The OOH* species formed over Au yield H_2_O_2_, whereas OOH* formed over Pd results in H_2_O due to facile OOH* scission over these monomers. An increased number of Pd monomers located at edge sites results in a substantial decrease in selectivity toward H_2_O_2_ as OOH* formation and subsequent O–O bond rupture over Pd is preferred, as compared to OOH* formation over Au.

The NP with a Pd monomer located both in the (111) facet and in an edge does not show increased selectivity compared to the NP with only one Pd monomer located in an edge. A large fraction of OOH* in this case is formed over Pd@Au(edge) rather than over Pd@Au(111). The net rate of H_2_O_2_ desorption from Pd is low, as OOH* dissociates easily over Pd@Au(edge), which is a consequence of a strong O_2_ adsorption energy. The preference of OOH* formation over Pd monomers, where the selectivity is low, is emphasized when an additional Pd monomer is added to the (100) facet. In this case, the steady state net rate of OOH* formation over Pd is similar to Au, whereas the net rate of H_2_O_2_ is low. The site communication between the different Pd monomers and the undercoordinated Au sites occurs mainly via water-mediated proton transfer. As the H^+^ diffusion in the solution is fast with respect to the surface reactions, the distance between the Pd monomers is not a crucial parameter in our simulations. Owing to the large differences in net formation rates of OOH* over the different Pd monomers, the selectivities over NPs with a larger number of Pd monomers are therefore determined by the worst-positioned Pd monomers that bind O_2_ strongly. Thus, the number of Pd monomers per NP should be kept low to obtain high selectivity. We elaborate on the influence of Pd monomer concentration in the SI.

#### General Observations

DFT calculations and kinetic Monte Carlo simulations have been used to explore direct H_2_O_2_ formation over Pd@Au alloy structures in an aqueous solution. The requirement for high selectivity toward H_2_O_2_ is that the catalysts should enable facile H_2_ dissociation and adsorption of O_2_. The interaction with O_2_ should not be too strong as to prevent irreversible O–O bond rapture.

We find that a selectivity close to 100% is obtained when a Pd monomer is embedded in the extended Au(111) surface. H_2_O_2_ is predominately formed via a mechanism where H^+^ is sequentially added to O_2_ via the water solution, rather than the conventional Langmuir–Hinshelwood mechanism. Thus, a crucial step for the high selectivity is the close to barrierless H^+^ desorption into the solution. The desorption process is a redox reaction where the electron is donated to the surface, whereas the proton forms a hydronium ion in the solution. The redox reaction is strongly exothermic thanks to the low adsorption energy of atomic H on Au surfaces and the high electronegativity of Au. Association of atomic H on the Au surface is unlikely at relevant temperatures, owing to the high recombination barrier. The reaction over Pd@Au(111) is strictly sequential, not only with respect to addition of protons to adsorbed O_2_ but also in the adsorption of H_2_ and O_2_. At least one of the H atoms from dissociated H_2_ must leave the Pd monomer before O_2_ can adsorb.

Technological implementation of Pd@Au systems requires Pd embedded in Au NPs. NPs are inherently different from extended surfaces thanks to the large number of different sites, including edge and corner sites. For example, 35% of the surface atoms of the studied 2.7 nm truncated octahedron are edges or corners. As O_2_ is known to adsorb on undercoordinated Au atoms, NPs enable additional reaction routes. With Pd embedded in Au NPs, it is possible to separate the sites for H_2_ and O_2_ adsorption. We have studied H_2_O_2_ formation over Pd monomers embedded in truncated Au octahedra. Placing the Pd monomer in the (111) facet of the NP results in a close to 100% selectivity toward H_2_O_2_; importantly, however, the reaction landscape and reaction mechanism is different from the extended Au(111) surface. The majority of H_2_O_2_ is formed over the NP edges, and the solution route is the dominating reaction mechanism also over NPs.

It is experimentally challenging to steer the location of the Pd monomers in the NPs. We find that the selectivity decreases to about 60% if the Pd monomer instead resides in a (100) facet or in an edge of the NP. The reason for the lower selectivity is a stronger adsorption energy of O_2_ to a low-coordinated Pd site. Thus, the efficiency of the site-separation for H_2_ and O_2_ adsorption is reduced. OOH* may form over undercoordinated Pd monomers, which also allow for facile OOH* dissociation. For NPs with multiple Pd monomers, we find that monomers with unfavorable positions determine the selectivity. The lower selectivity over ill-positioned Pd monomers can to some extent be compensated by tuning the reaction conditions. An increased *p*(H_2_)/*p*(O_2_) ratio increases the selectivity toward H_2_O_2_, which is in agreement with experimental results.^18^ The higher selectivity originates from H blocking of the Pd monomer, which steers the OOH* formation to undercoordinated Au sites. Our simulations reproduce the experimentally reported^18^ dependence on H_2_ and O_2_ pressure on H_2_O_2_ formation rate (see SI). The reaction order in H_2_ is positive at low pressures and close to zero at high pressures, whereas the reaction order in O_2_ is slightly negative in the investigated pressure interval.

The apparent activation energies for H_2_O_2_ and H_2_O formation over an ensemble of NPs have experimentally been reported to be 0.18 and 0.75 eV, respectively.^18^ A direct comparison to our results is difficult due to the strong dependence on the number and placement of Pd monomers in the Au NPs; however, Pd positioned in the extended Au(111) could be a reasonable representation. We calculate the apparent activation energies for H_2_O_2_ and H_2_O formation over Pd@Au(111) to be 0.38 and 0.73 eV, respectively. The apparent activation energy for H_2_O_2_ formation over a Au NP with Pd positioned in a Au(111) facet is also 0.38 eV. The apparent activation energies for both H_2_O_2_ and H_2_O formation increase when Pd is placed in undercoordinated positions. The difference in apparent activation energies between H_2_O_2_ and H_2_O is primarily related to the competing H_2_O_2_ formation and OOH* dissociation, which may occur on different sites. We find that the H_2_ dissociation determines the rate of the reaction, as close to all H on the catalyst surface react to form either H_2_O_2_ or H_2_O.

## Conclusions

We have developed a first-principles-based kinetic Monte Carlo approach to explore the governing factors in direct formation of H_2_O_2_ over Pd embedded in Au NPs and extended surfaces in a water solution. Whereas a negligible amount of H_2_O_2_ is formed over pure Au NPs, systems with Pd monomers embedded in Au are active for the reaction. We find that a high activity and selectivity is possible over dilute Pd@Au NPs thanks to an efficient site separation, where H_2_ dissociation occurs over Pd sites and O_2_ adsorption and hydrogenation occur over Au edges and corners. Atomic H on Au is found to undergo a redox reaction where H^+^ desorbs to the water solution, leaving an electron in the metal. The water solution is found to mediate the reaction by enabling facile H^+^ diffusion. OOH and H_2_O_2_ are predominantly formed by reactions between O_2_ and OOH and dissolved H^+^. The simulations stress the need to account for the complete potential energy landscapes of the solvated NPs to properly describe the kinetic behavior of the reaction. Our results rationalize experimental findings and provide guidelines to design and operate single atom alloy catalysts to obtain high activity and selectivity for a range of different hydrogenation reactions.

## Supporting Information Available

Constrained molecular dynamics simulations for H^+^ diffusion; analysis of energy contributions in the formation of hydronium ions in the water solution; barriers for H_2_ dissociation and OOH and H_2_O_2_ formation over the considered surfaces; data for scaling relations; influence of the number of Pd monomers in the kMC simulations; TOF as a function of partial pressures; analysis of how the charge state of adsorbed O_2_ depends on the presence of water (PDF)
