# Supplementary material for: Site Communication in Direct Formation of H2O2 over Single-Atom Pd@Au Nanoparticles
Source: J Am Chem Soc. 2023 May 16;145(21):11579–88. doi: 10.1021/jacs.3c00656 (PMC10236495; doi:10.1021/jacs.3c00656)
Supplement: Supplementary file 1 — ja3c00656_si_001.pdf [file ja3c00656_si_001.pdf]

Supporting Information: Site Communication in Direct Formation of H_2O_2 over Single-Atom Pd@Au Nanoparticles

Rasmus Svensson* and Henrik Grönbeck*

Department of Physics and Competence Centre for Catalysis, Chalmers University of Technology, SE-412 96 Göteborg, Sweden

E-mail: rassve@chalmers.se; ghj@chalmers.se

Proton Transfer - Constrained MD simulations

The redox-reaction, where a proton is transferred to the water solution and an excess electron is left on the metal surface is studied using constrained molecular dynamics, see Figure S1. The simulations reveal that the process is close to barrierless over Pd@Au(111) and Au(111), whereas the process is associated with a barrier of about 0.5 eV over Pd(111). The process is highly exothermic over Pd@Au(111) and Au(111), whereas it is slightly endothermic over Pd(111).

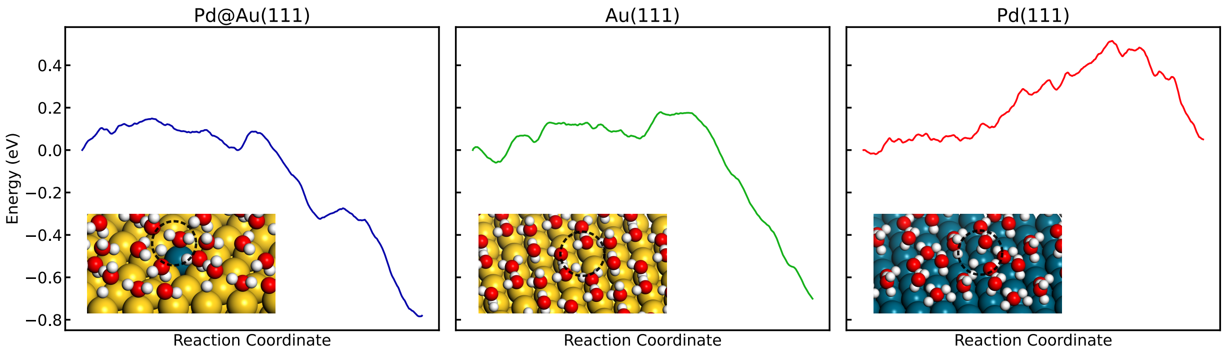

Figure S1: Constrained molecular dynamics simulations for proton transfer from the metal to the water solution over Pd@Au(111), Au(111) and Pd(111). To reduce the noise from O−H vibrations, each step in the trajectory is a running average over 600, 800 and 600 steps, for Pd@Au(111), Au(111) and Pd(111), respectively.

A simple decomposition of the energy contributions is made to elucidate the factors governing the exothermicity of the H^+ transfer to the solution. A schematic is shown in Figure S2 considering the case with complete charge separation to H^+ and e^-.

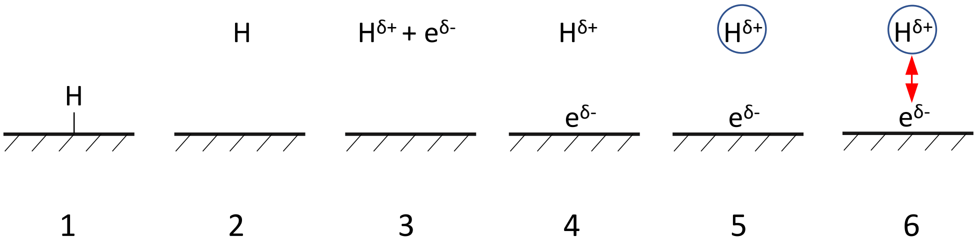

Figure S2: Schematic of the energy contributions for the redox-reaction where a proton is transferred to the water solution and an excess electron is delocalized over the metal surface.

1) H adsorbed on the surface (reference value).

$$E_1 = 0 \text{ eV} \tag{1}$$

2) Desorption of atomic H.

$$E_2 - E_1 = 2.11 \text{ eV} \tag{2}$$

3) Ionization of H.

$$E_3 - E_2 = E_{\text{ionization}} = 13.6 \text{ eV} \tag{3}$$

4) Electron transfer to surface.

$$E_4 - E_3 = \phi \times \delta = -5.30 \text{ eV} \tag{4}$$

5) Solvation of the proton in water.[S1]

$$E_5 - E_4 = E_{\text{solvation}} \approx -11 \text{ eV} \tag{5}$$

6) Coulomb interaction (point charge assumed)

$$E_6 - E_5 = -k \frac{1}{4\pi\varepsilon_0 d} \delta^2 \tag{6}$$

Here, ϕ is the work-function of the metal, δ is the charge transferred in the process (1 electron), d is the distance between the surface and the proton and k is a permittivity scaling due to the water molecules. The reaction energy without the Coulomb interactions is

$$E_5 - E_1 = -0.59 \text{ eV} \tag{7}$$

The potential energy owing to the Coulomb interactions is difficult to estimate, because of the presence of water. However, k should be somewhere between zero and one. Therefore, with $d = 5\,\text{Å}$, the total energy difference is between negative 0.6-2 eV. The deconvolution of the energies indicate that the exothermicity of the reaction can be traced to the low adsorption energy of H on Au and the high work function of Au. The difference between Au(111) and Pd(111) is roughly the difference in the adsorption energy of H over the two surfaces, as the work functions for the two metals are similar.

Additional Energies

H_2 adsorption energy together with H_2 dissociation and association barriers over Pd@Au(100) and Pd@Au(211) are reported in Table S1.

Table S1: H_2 adsorption energies (E_{ads}), H_2 dissociation barrier ($E_{\mathrm{a,f}}$) and association barrier ($E_{\mathrm{a,b}}$) over Pd@Au(100) and Pd@Au(211).

Surface	Reaction Equation	E_{ads} (eV)	$E_{\mathrm{a,f}}$ (eV)	$E_{\mathrm{a,b}}$ (eV)
Pd@Au(100)	$H_2 + {}^* \rightleftharpoons {H_2}^*$	-0.308	-	-
Pd@Au(211)	$H_2 + {}^* \rightleftharpoons {H_2}^*$	-0.329	-	-
Pd@Au(100)	${H_2}^* + {}^* \rightleftharpoons 2H^*$	-	0.326	0.422
Pd@Au(211)	${H_2}^* + {}^* \rightleftharpoons 2H^*$	-	0.341	0.420

The potential energy surfaces presented in the main text show the H_2O_2 formation route where protons react with ${O_2}^*$ and OOH^* via the water solution. The corresponding reactions, where surface-bound H^* reacts with ${O_2}^*$ and OOH^* (Langmuir-Hinshelwood mechanism) are shown in Table S2.

Table S2: Energy differences and energy barriers for the addition of hydrogen to O_2 and OOH forming OOH and H_2O_2, over Pd@Au(111) and Pd@Au(211) according to the Langmuir-Hinshelwood pathway. $E_{\mathrm{a,f}}$ and $E_{\mathrm{a,b}}$ denote the forward and backward energy barriers, respectively.

Surface	Reaction Equation	ΔE (eV)	$E_{\mathrm{a,f}}$ (eV)	$E_{\mathrm{a,b}}$ (eV)
Pd@Au(111)	${O_2}^* + H^* \rightleftharpoons OOH^* + {}^*$	-0.791	0.614	1.405
Pd@Au(111)	$OOH^* + H^* \rightleftharpoons H_2{O_2}^* + {}^*$	-0.883	0.319	1.202
Pd@Au(211)	${O_2}^* + H^* \rightleftharpoons OOH^* + {}^*$	-0.758	0.559	1.317
Pd@Au(211)	$OOH^* + H^* \rightleftharpoons H_2{O_2}^* + {}^*$	-0.274	0.352	0.626

Scaling Relations

To describe the potential energy surface over the range of under-coordinated Au sites on the NP, scaling relations are used. The adsorption energy is determined using generalized coordination numbers as a descriptor.[S2,S3] The data used to obtain the scaling relations are shown in Table S3. O_2, OH and OOH are preferably adsorbed at bridge sites, whereas O is adsorbed either at bridge or hollow sites. The adsorption energies are given with respect to the bare surface and gas phase H_2 and O_2.

Table S3: Energies used to obtain the scaling relations over the Au NPs. For each structure and adsorption site, the non-zero-point corrected adsorption energy, as well as the coordination number and generalized coordination number are presented.

Structure	Adsorption Site	CN	GCN	$E_{ads}(O)$ (eV)	$E_{ads}(OH)$ (eV)	$E_{ads}(OOH)$ (eV)	$E_{ads}(O_2)$ (eV)
111	Bridge	13	22/3	-	-1.4028	-0.6859	-
111	fcc-hollow	15	153/22	-0.0725	-	-	-
100	Bridge	12	20/3	-0.07314	-1.8678	-0.9839	-0.0743
211	Bridge	10	97/18	-0.2088	-1.9509	-1.0247	-0.3140
Defected 211	Bridge	11	109/18	-	-	-	-0.1798
532(1)	Bridge	9	89/18	-	-	-	-0.3683
532(2)	Bridge	10	11/2	-	-	-	-0.2830
2Ad 100	Bridge	6	28/9	-0.2245	-1.9492	-1.0641	-
2Ad 111	Bridge	5	17/6	-0.4554	-2.2185	-1.3819	-
3Ad 111	Bridge	6	19/6	-	-2.0121	-1.2261	-
3Ad 111	hcp-hollow	7	36/11	-0.3901	-	-	-
KINKED 111	Bridge	12	13/2	-	-1.7941	-1.0345	-
KINKED 111	fcc-hollow	14	137/22	-0.3317	-	-	-
KINKED 211	Bridge	8	79/18	-0.1325	-1.9640	-1.1271	-

Influence of the Number of Pd Monomers

To elucidate how the Pd@Au composition affects the TOF and selectivity in further detail, the number of Pd monomers in the structures are increased. The number of Pd monomers in the Au(111) surface is increased from one to eight (0.25 % to 2 %). Similarly, the number of Pd monomers, either in different (111) facets or in edges in the NP, is increased from one to eight (0.37 % to 2.9 %). The TOF and selectivity as a function of the number of Pd monomers are shown in Figure S3. When Pd i located in the (111) surface or in the (111) facets of the NP, the TOF and selectivity decreases only slightly with an increased number of Pd monomers. However, when the concentration of Pd monomers embedded in edges increases, the selectivity is significantly reduced, from around 66 % to 14 %. This is owing to the strong adsorption energy of O_2 and facile OOH^* dissociation over Pd@NP(edge). In conclusion, the number of Pd monomers in the NP can be increased, as long as the monomers are located in Au(111) facets.

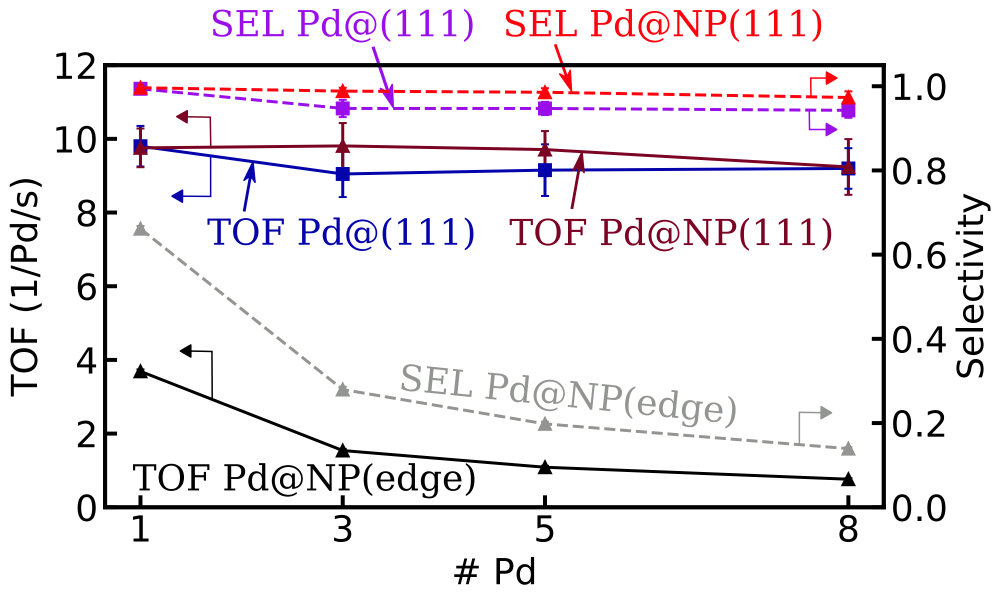

Figure S3: The turn-over frequency and selectivity over Pd@Au(111) and Pd located only in the Au(111)-facets or only in the edges of the NP. All simulation are performed at 286 K and 100 kPa O_2 and H_2 pressure. The standard deviations are determined from 30 independent simulations.

TOF as a Function of Partial Pressures

The number of Pd monomers per NP is experimentally not restricted to one. Therefore, to facilitate comparison between our results and experimental results, a truncated Au octahedron with eight Pd monomers located at different (111)-facets are used. The turn-over frequency, as a function of H_2 pressure (left) and O_2 pressure (right) are shown in Figure S4. At low H_2 pressures, the TOF scales linearly with the H_2 pressure. However, at higher pressures, the TOF reaches a plateau. The coverage of H/H_2 over the Pd monomer is then approaching one. Thus, the reaction order in H_2 is strongly positive at low H_2 pressures, whereas it approaches zero for high pressures. This trend is in excellent agreement with experimental results.[S4] The exact pressure, for which the plateau is reached, is exponentially dependent on the adsorption energies and might be overestimated in the DFT calculations. If the O_2 pressure is increased, at a constant H_2 pressure, the TOF decreases slightly. The decreased TOF is a consequence of the O_2 blocking the Pd monomer from adsorbing and dissociating H_2. The decreasing trend is also in agreement with the experimental results.[S4]

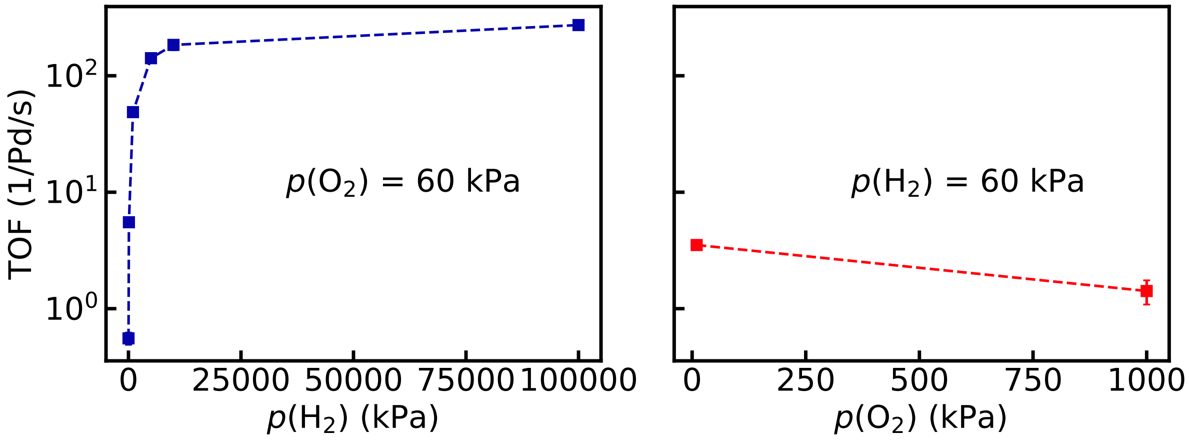

Figure S4: The turn-over frequency over a 2.7 nm truncated Au octahedron with eight Pd monomers located in different (111)-facets [8 Pd@NP(111)], as a function of left) H_2 pressure (at 60 kPa O_2 pressure) and right) O_2 pressure (at 60 kPa H_2 pressure). All simulations are performed at 276 K and the standard deviations are determined from 16 independent simulations.

The Influence of Surface Charge and Water on O_2 Adsorption Energy

Over a bare Pd@Au(111) surface (no water), the Pd monomer is calculated to have a positive Bader charge of 0.06e. The influence of charge is investigated for difference surface cells, Table S4. The charge in the metal surface is in this case added artificially by adding one electron to the system. All surface structures are four layers thick. The non-zero-point corrected adsorption energies of O_2, as well as the O-O bond lengths and O_2 Bader charges are shown. The adsorption energy of O_2 stays relatively constant regardless of cell size and surface charge.

Table S4: The O_2 adsorption energy, O-O bond length and O_2 Bader charge upon adsorption, for a range of different Pd@Au(111) cell sizes and charges.

Size	Surface Charge (e)	$E_{ads}(O_2)$ (eV)	O-O Bond Length (Å)	O_2 Bader Charge (e)
2x2	0	-0.38	1.267	0.24
2x2	-1	-0.47	1.293	0.45
3x3	0	-0.34	1.267	0.23
3x3	-1	-0.34	1.280	0.35
3x3	-2	-0.44	1.296	0.47
4x4	0	-0.37	1.281	0.30
4x4	-1	-0.38	1.293	0.37
5x5	0	-0.36	1.281	0.30
5x5	-1	-0.36	1.286	0.34

The adsorption energy of O_2 is also relatively unchanged over a 116 atom truncated Au octahedron, regardless whether the surface is charged or not. DFT calculations are performed for the structure shown in Figure S5. The non-zero-point corrected adsorption energy of O_2 is over the uncharged structure $-0.44\,\text{eV}$. When an additional electron is introduced to the truncated octahedron structure, the adsorption energy of O_2 is virtually unchanged ($-0.45\,\text{eV}$).

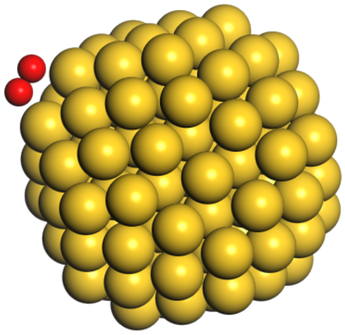

Figure S5: The O_2 adsorption on the 116 atom truncated Au octahedron.

A water layer above the Au surface has clear effects on the adsorption energy and charge state of O_2. To investigate the effect of water, DFT calculations are performed over five different Pd@Au(111) structures in a (3x3) surface cell with four atomic layers. The results are shown in Table S5.

Table S5: The O-O bond length as well as the O_2 Bader charge for O_2 adsorption over neutral and charged Pd@Au(111) with and without a water layer.

Structure	O-O Bond Length (Å)	O_2 Bader Charge (e)
Pd@Au(111) (no water)	1.267	0.23
Pd@Au(111) (no water + e^-)	1.280	0.35
Pd@Au(111) (water)	1.305	0.48
Pd@Au(111) (water + H^+ + e^-)	1.323	0.61
Pd@Au(111) (water + e^-)	1.323	0.62

The O-O bond length and O_2 Bader charge is significantly increased when water is included in the calculations. However, an excess electron has only a slight effect on the adsorption configuration. In water, O_2 is negatively charged when adsorbed on the Pd monomer, *i.e.*, the surface is positively charged. Upon the proton-transfer to the water solution, 0.65e is donated to the surface/adsorbate structure. Only a small part of this charge is located on the adsorbed O_2 molecule; most of the electron is delocalized over the surface.

References

(S1) Tawa, G.; Topol, I.; Burt, S.; Caldwell, R.; Rashin, A. Calculation of the aqueous solvation free energy of the proton. *J. Chem. Phys.* **1998**, *109*, 4852–4863.

(S2) Calle-Vallejo, F.; Martínez, J. I.; García-Lastra, J. M.; Sautet, P.; Loffreda, D. Fast prediction of adsorption properties for platinum nanocatalysts with generalized coordination numbers. *Angew. Chem. Int. Ed.* **2014**, *53*, 8316–8319.

(S3) Calle-Vallejo, F.; Tymoczko, J.; Colic, V.; Vu, Q. H.; Pohl, M. D.; Morgenstern, K.; Loffreda, D.; Sautet, P.; Schuhmann, W.; Bandarenka, A. S. Finding optimal surface sites on heterogeneous catalysts by counting nearest neighbors. *Science* **2015**, *350*, 185–189.

(S4) Ricciardulli, T.; Gorthy, S.; Adams, J. S.; Thompson, C.; Karim, A. M.; Neurock, M.; Flaherty, D. W. Effect of Pd coordination and isolation on the catalytic reduction of O2 to H2O2 over PdAu bimetallic nanoparticles. *J. Am. Chem. Soc.* **2021**, *143*, 5445–5464.
